# Structural proteomics reveals the functional docking interface of ferredoxin‐NADP ^+^ reductase on photosystem I in the red alga *Cyanidioschyzon merolae*

**DOI:** 10.1111/tpj.71017

**Published:** 2026-07-01

**Authors:** Muhammad Younas, Yuval Milrad, André Vidal‐Meireles, Samuel Wink, Karen Zinzius, Martin Scholz, Michael Hippler

**Affiliations:** ^1^ Institute of Plant Biology and Biotechnology University of Münster Münster 48143 Germany; ^2^ Institute of Plant Science and Resources Okayama University Kurashiki Okayama 710‐0046 Japan; ^3^ Present address: CEA, CNRS, BIAM, UMR7265 Aix Marseille University Saint‐Paul‐Lez‐Durance France

**Keywords:** photosynthesis, electron transfer, mass spectrometry, cross‐linking, ferredoxin‐NADP+ reductase, photosystem I

## Abstract

Efficient photosynthetic electron transfer relies on transient interactions between photosystem I (PSI) and soluble electron carriers. However, structural information describing these transient interactions are limited in red algae. Here, we applied chemical cross‐linking mass spectrometry (XL‐MS) to isolated thylakoid membranes of the red alga *Cyanidioschyzon merolae* and generated an interactome map of photosynthetic protein complexes. Using three independent cross‐link identification algorithms, we obtained a high‐confidence dataset of intra/intercomplex interactions. Among them, we identified a cross‐link between K94 of FNR and K108 of the stromal subunit PsaD. Cross‐linking restraint‐guided protein–protein docking using HADDOCK2.4 revealed a direct docking interface for FNR on PsaD side. Structural analysis of the resulting complex indicated that the binding of FNR to PSI is predominantly electrostatic, in which the K4 residue of FNR is involved in making a salt bridge with E91 of PsaD as well as a conventional hydrogen bond with G90 of PsaD. Site‐directed mutagenesis of the FNR K4 residue significantly impaired the NADP^+^ reduction kinetics, as compared with the WT FNR, supporting that FNR binding to PSI is required for efficient FNR and ferredoxin (FDX)‐dependent NADP^+^ photoreduction. These results strongly suggest that FNR binds to the stromal side of PSI in an orientation that enables efficient electron transfer from FDX. Additionally, we detected a cross‐link between the phycobilisome core protein ApcA and PsaD suggesting that it may serve as a shared interaction hub for both FNR and PBS, implying that these interactions could either occur simultaneously or compete for binding while still allowing electron transfer via soluble FDX.

## Significance statement

Through structural proteomics and molecular modeling, we identified a novel docking site for ferredoxin‐NADP^+^ reductase on Photosystem I. Site‐directed mutagenesis reveals that this electrostatic interface is vital for efficient electron transfer in *Cyanidioschyzon merolae*, offering new insights into the structural basis of optimized photosynthetic energy conversion in red algae.

## INTRODUCTION

Oxygenic photosynthesis sustains life on Earth by converting light energy into chemical energy through protein complexes in the thylakoid membranes. The process begins with light‐driven charge separation at photosystem II (PSII), initiating linear electron flow where electrons from water photolysis transfer through plastoquinone to the cytochrome *b*_*6*_ *f* complex (Cyt*b*_*6*_ *f*). In the photosynthetic domain, two soluble electron carriers mediate the transmission between Cyt*b*_*6*_ *f* and photosystem I (PSI), namely: plastocyanin (PC) and cytochrome *c*_*6*_ (Cyt*c*_*6*_), depending on phylogenetic lineage and/or environmental availability of substances such as copper or iron (Milrad et al., 2024; Slater et al., 2021). The evolution of these proteins, as well as their interaction mechanisms with both PSI and cytochrome *f*, have been extensively investigated (De La Rosa et al., 2002; Hervás et al., 1997; Navarro et al., 1997). Following its reduction, PSI absorbs light and excites the electrons to high potential, which then reduces ferredoxin (FDX). FDXs deliver electrons to various pathways, most prominently, they reduce ferredoxin‐NADP(+) reductase (FNR) which facilitates the reduction of NADP^+^ to NADPH, a central substance of sugar synthesis, in the process of linear electron flow (LEF). In addition to FDX, the non‐iron protein flavodoxin can serve as an alternative electron acceptor for PSI in many photosynthetic organisms, particularly under iron‐limiting conditions (Goñi et al., 2009; Pierella Karlusich & Carrillo, 2017). However, no flavodoxin‐dependent PSI electron transfer pathway has so far been identified in *C. merolae* and, therefore, the present study focuses on the FDX‐dependent PSI‐FNR interaction. FNR was shown to play a major role in electron flow regulation, based on its availability and spatial localization (Kramer et al., 2021). Although classically considered to be a soluble stromal enzyme, in vascular plants, it was shown to be anchored to the thylakoid membranes near PSI via proteins such as: thylakoid rhodanase‐like protein (TROL) and translocon at the inner envelope of chloroplasts 62 (Tic62) (Rodriguez‐Heredia et al., 2022). However, recent studies suggest that in green algae, FNR forms transient complexes with PSI‐LHCI supercomplexes independently of any additional peptidal anchoring (Marco et al., 2019; Mosebach et al., 2017; Takahashi et al., 2014), optimizing spatial organization by minimizing diffusion and increasing reaction specificity. Moreover, spatial‐based competition was shown to play a major kinetic role in hydrogen production studies of green algae under anaerobic conditions. Accordingly, the localization of FNR in the proximity of PSI grants it a kinetic advantage over other processes, supplementing for its overall lower specific activity (Eilenberg et al., 2016; Yacoby et al., 2011). This dynamic association, influenced by environmental conditions and metabolic demand, highlights FNR as a semi‐soluble component coordinating NADPH production, though molecular mechanisms governing these interactions require further investigation.

The red alga *Cyanidioschyzon merolae* serves as a valuable model for studying photosynthetic evolution. Having a relatively simple genomic and cellular architecture, makes it a suitable organism for subcellular studies (Miyagishima & Tanaka, 2021). Moreover, in regard to photosynthetic research, its PSI complex was shown to possess light‐harvesting complexes (LHC), that are homologous to both PSI and PSII antennae in green lineages. Yet, unlike other eukaryotic photosynthesizers, it retained the phycobilisome (PBS) antenna systems, which makes it an ideal intermediate between them and the primal cyanobacteria (Busch et al., 2010; Stadnichuk & Kusnetsov, 2023). On the other hand, *C. merolae* was shown to lack subunits PsaG and PsaH (Matsuzaki et al., 2004), which are present in green algae and plants. Other interesting features, which attracted many studies, are their ability to thrive in extreme environments such as acidic hot springs and their exclusive usage of Cyt*c*_*6*_ as a luminal electron carrier. However, molecular details of PSI electron transport in *C. merolae*, particularly interactions with redox partners such as Cyt*c*_*6*_, FDX and FNR, remain incompletely understood. In this work, we examined the interactions between PSI and its associated redox partners. By cross‐linking isolated thylakoid membranes, we managed to generate an interface map of stromal PSI. We present a homologous interaction with FDX, while proposing a new docking site for FNR, placing it on PsaD and discuss its role in regulating photosynthetic electron transfer. Moreover, we also shed light on the potential association of PBS with PSI as well as an insight into the previously unresolved loop in PsaE subunit of PSI in *C. merolae*.

## RESULTS

### Photosystem I interactome map

**Figure 1:**
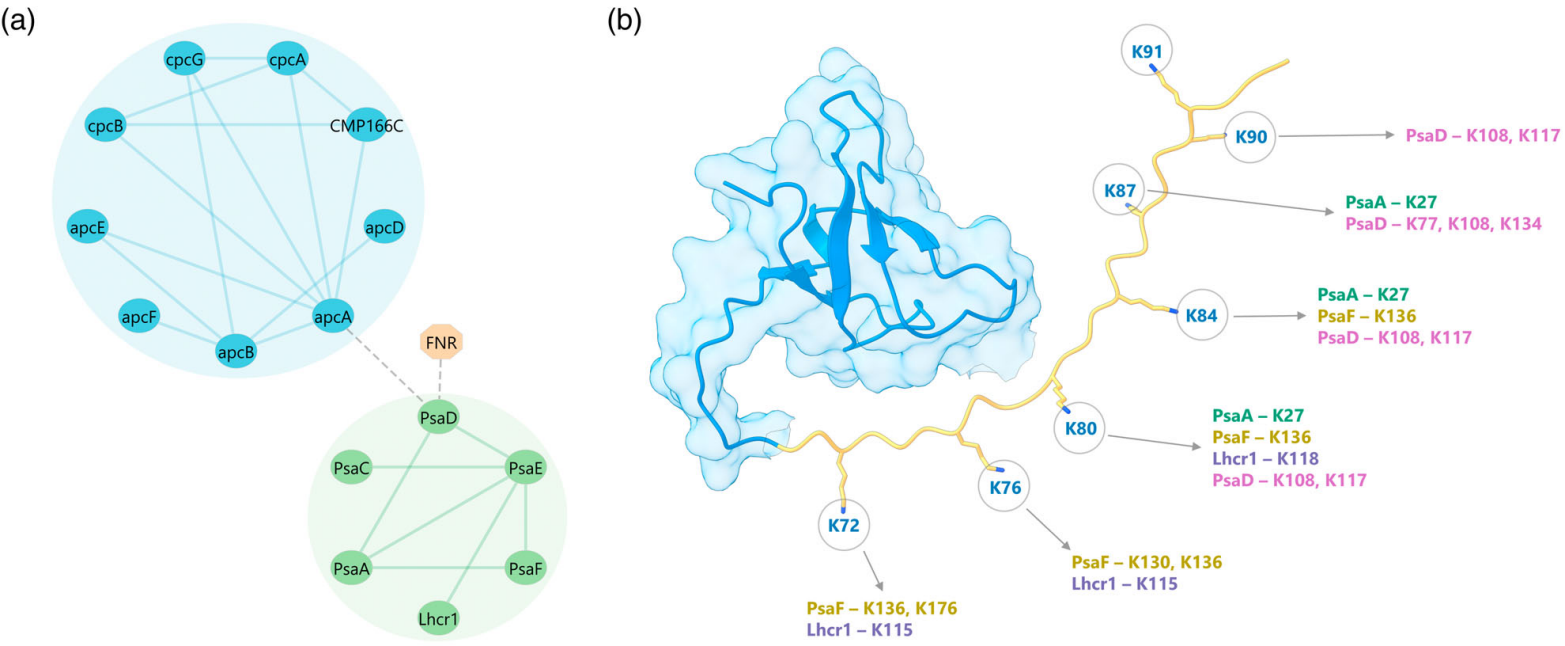
XL‐MS‐based interactome and structural modeling of the PSI complex. (a) Protein–protein interaction network derived from cross‐linking mass spectrometry (XL‐MS) data. Nodes represent subunits of the phycobilisome (blue) and Photosystem I (green); edges indicate detected cross‐links. The orange node denotes Ferredoxin‐NADP+ reductase (FNR). (b) Structural mapping of cross‐links onto the PsaE subunit. The previously unresolved loop is highlighted in gold. Labeled lysine (K) residues indicate the sites of cross‐links to neighboring subunits (e.g., PsaA, PsaF, Lhcr1, and PsaD), with specific interaction partners and their respective residues indicated by arrows.

To gain insight into the photosynthetic protein–protein interactions (PPIs) in *C. merolae*, the isolated thylakoid membranes were cross‐linked using the amine‐reactive crosslinker DSS (Mosebach et al., 2024), followed by solubilization with three different detergents (α‐DM, β‐DM, and OG). The solubilized membranes were then subjected to sucrose density gradient fractionation, followed by tryptic digestion and subsequent identification of the cross‐linked peptides using LC–MS/MS analysis (Albanese et al., 2020; Piersimoni et al., 2022). Cross‐links identification was done using three different algorithms including MaxQuant, XiSearch, and pLink 2. Extensive set of inter/intra‐protein cross‐links were detected involving the major photosynthetic protein complexes including PSII, PSI, Cyt*b*_*6*_ *f*, ATP synthase, and PBS as well as several assembly factors. In total, more than 700 unique residue pairs were detected across the dataset (Supplementary File 1). To increase confidence, only those cross‐links that were independently identified by all three search algorithms (MaxQuant, XiSearch, and pLink) were retained for further analysis. This filtering resulted in a high‐confident dataset comprising 223 unique residue pairs. Of these, 95 correspond to interprotein cross‐links between subunits of major photosynthetic protein complexes, which include interactions within PBS (32), PSI (34), PSII (7), ATP synthase (10), as well as several intercomplex cross‐links including PBS–PSII (2), PBS–PSI (1), PSI–FNR (1), and one involving the Cyt*b*_*6*_ *f*. The identified cross‐links were then mapped as a network using Cytoscape (Figure S1) (Shannon et al., 2003). While this dataset provides a comprehensive cross‐linking‐based interaction map of the entire photosynthetic apparatus, subsequent analysis focused on PSI‐associated interactions, and so we generated an interactome map for PSI in *C. merolae* (Figure 1a).

A prominent feature of this interactome map (Figure 1a) is the identification of a cross‐link between the core allophycocyanin subunit apcA of PBS and the stromal subunit PsaD of PSI (apcA:K27–PsaD:K108). This PBS‐PSI cross‐link was observed in the thylakoids isolated from both 25°C and 42°C grown cultures of *C. merolae* (Supplementary File 1). Structural analysis based on the available PSI structure of *C. merolae* indicated that the cross‐linked PsaD residue (K108) is located on the stromal side of PSI, suggesting that the detected cross‐link is structurally feasible. While structural information for the PBS from *C. merolae* is for the moment unavailable, indirect information about its organization can be inferred from structural studies of cyanobacteria and other red algae (Bryant & Gisriel, 2024; Li et al., 2023; Zhang et al., 2017). A typical PBS consists of an allophycocyanin core, which is further composed of 2, 3, or 5 cylinders of (αβ)3 allophycocyanin trimers, from which several radiating rods extend. These rods are built from stacks of hexamers that are formed by phycocyanin αβ protomers and, in some cyanobacteria and red algae, phycoerythrin (Bryant et al., 1981; Bryant & Gisriel, 2024; Domínguez‐Martín et al., 2022; Ma et al., 2020; Zheng et al., 2021, 2025). However, unlike other red algae, phycoerythrin is absent in *C. merolae* (Krupnik et al., 2023).

### Using the PsaE subunit as a “fishing hook”

Looking through our dataset, we observed numerous cross‐links related to a loop region of PsaE subunit (Supplementary File 1; Figure 1b). Strikingly, many of these events were located to a structurally unresolved C′ loop. To further investigate this loop, we aligned all the available PsaE protein sequences from the Uniprot database (search filters was set to either: gene or protein containing the word: “PsaE,” and 1236 results were gathered for final comparison, see Supplementary File 2). We compared the sequences of all oxygenic photosynthesizers and observed that while members of the green lineage have no elongated C′ tail, many members of the Bangiophyceae, a class of Rhodophytes which includes the order Cyanidiales, have a long, very positively charged tail. In fact, in most cases where we did find a prolonged tail, it was positively charged by lysine residues (Figure S3). In the red alga *C. merolae,* we measured the tail to contain 33 amino acids, 9 of which are lysines, that sum to around 25% of its residues. The orientation of this wobbly region was not yet resolved in any structural dataset (PDB IDs: 5ZGH, 5ZGB [Pi et al., 2018], 7BLZ [Nelson, 2024], and 6FOS [Antoshvili et al., 2019]). Moreover, *in silico* computations using AlphaFold 3 (Abramson et al., 2024) did not result in a reliable structural assessment (Figure 1b). This could be explained by its wobbliness, a feature that is not optimal for current structural methodologies. However, such a region often serves as a hook that fish out relevant soluble complexes, especially when it is robustly charged. Here, based on our XL‐MS data, it was shown forming contact with nearby PSI core subunits (Figure 1b, see also Supplementary File 1). Notably, several cross‐links connected this loop to the PSI‐associated antenna protein (Lhcr1), which indicates that this loop may occupy a different spatial position relative to the core of PSI. Specifically, PsaE:K72 and K76 were found cross‐linked to K115 of the PSI‐associated light‐harvesting antenna (Lhcr1) whereas PsaE:K80 was cross‐linked to Lhcr1:K118. Together, these observations identify this unresolved loop of PsaE as a highly interactive and flexible region of PSI in *C. merolae*, perhaps attributing it a yet unidentified function.

### FNR–PSI interaction and its functional validation

**Figure 2:**
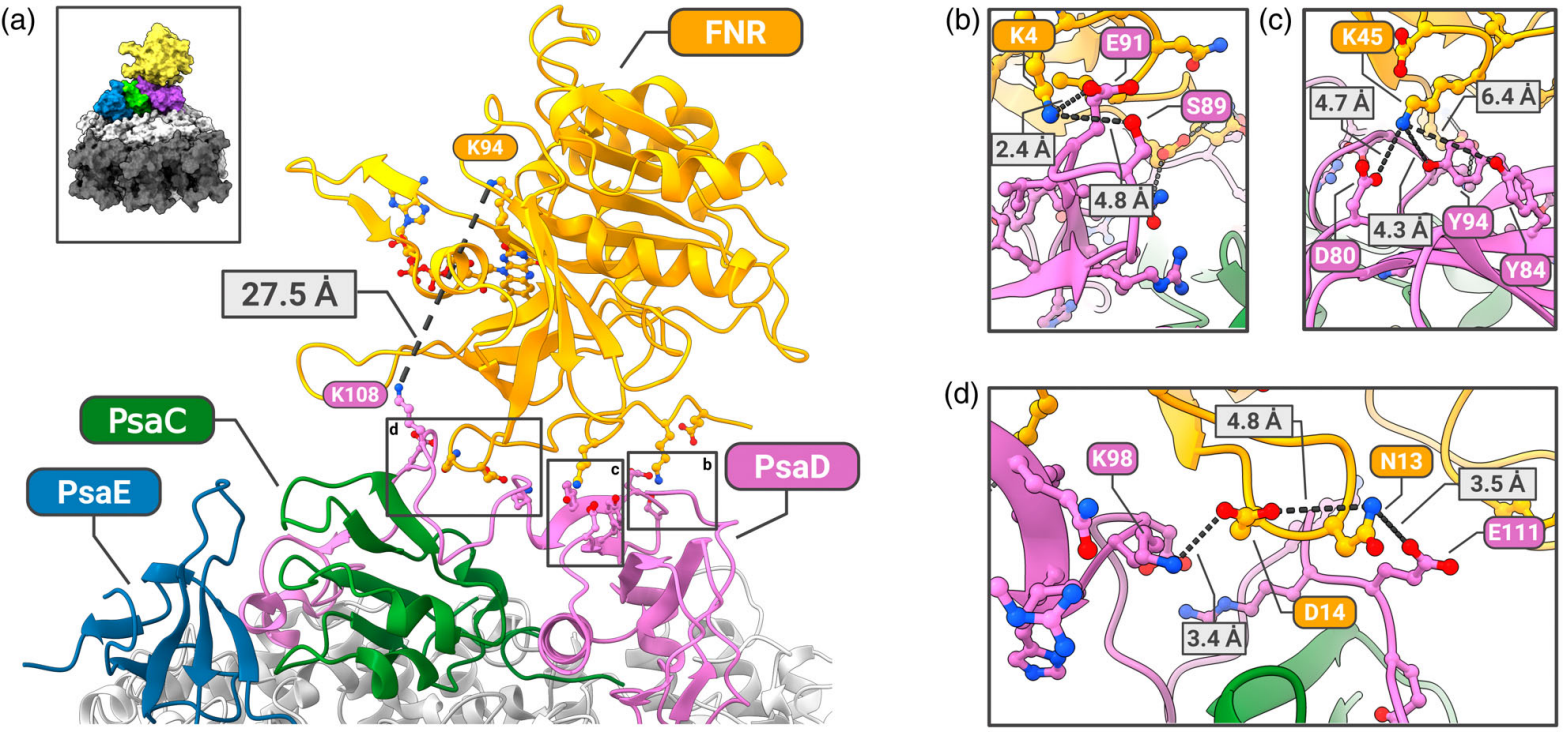
Structural model of the FNR–PSI complex based on XL‐MS constraints. (a) Representative docking pose of FNR (yellow) on the PSI stromal subunits PsaC (green), PsaE (blue), and PsaD (pink), generated using HADDOCK v2.4. The Euclidean distance between N‐N atoms of cross‐linked lysine residues (K94 of FNR and K108 of PsaD) is indicated (27.5 Å). Inset: Overview of the FNR–PSI assembly within the membrane‐bound complex. (b–d) Detailed views of the FNR–PsaD interface. Dashed lines represent predicted atomic‐level interactions, with inter‐residue distances highlighted. Specific residues involved in the stabilization of the complex, including FNR (yellow labels) and PsaD (pink labels), are shown in stick representation. The figure was generated using ChimeraX and BioRender.com.

In addition to PBS interactions, another prominent feature of the PSI interactome map (Figure 1) is the identification of a cross‐link between FNR and PSI. Our results clearly show a cross‐link between FNR:K94 and PsaD:K108. To evaluate the structural compatibility of FNR–PSI association, the AlphaFold‐generated model of FNR (UniProt entry nr: M1V6N7, transit peptide predicted with TargetP 2.0 (Almagro Armenteros et al., 2019) and removed from final protein) was docked to the available PSI structure of *C. merolae* based on cross‐linking data using the HADDOCK2.4 server (Honorato et al., 2024). To generate a model, based on our cross‐linking data, we restricted the interactive distances between FNR:K94 and PsaD:K108 using DSS compatible distance threshold (25–30 Å). The best model was selected from the list of generated models based on HADDOCK scoring. The resulting model positioned the FNR on the stromal surface of PSI in a configuration that satisfies both the cross‐link and show no clashes between residues (Figure 2a, for the full model, please see Supplementary File 3). Interestingly, analysis of the docked interface revealed that the binding of FNR to PSI in the hypothetical model is mostly electrostatic, where multiple salt bridges, hydrogen bonds as well other kind of interactions such as Pi‐Cation, Pi‐Alkyl, and carbon–hydrogen bonds stabilizes the interaction. To further investigate this, we calculated potential interactions in model using LigPlot (Laskowski & Swindells, 2011) and saw strong potential binding between FNR and PsaD (Figure S2). Most interestingly was FNR:K4, which seems to be pocketed between E91 and S89 (and the backbone oxygen of G90) of PsaD (Figure 2b). In addition, we observed potential interactions surrounding FNR:K45, with PsaD:D80,Y94 and Y84 (Figure 2c) and the duo FNR:N13‐D14, that interact with PsaD:E111 and K98, respectively (Figure 2d).

**Figure 3:**
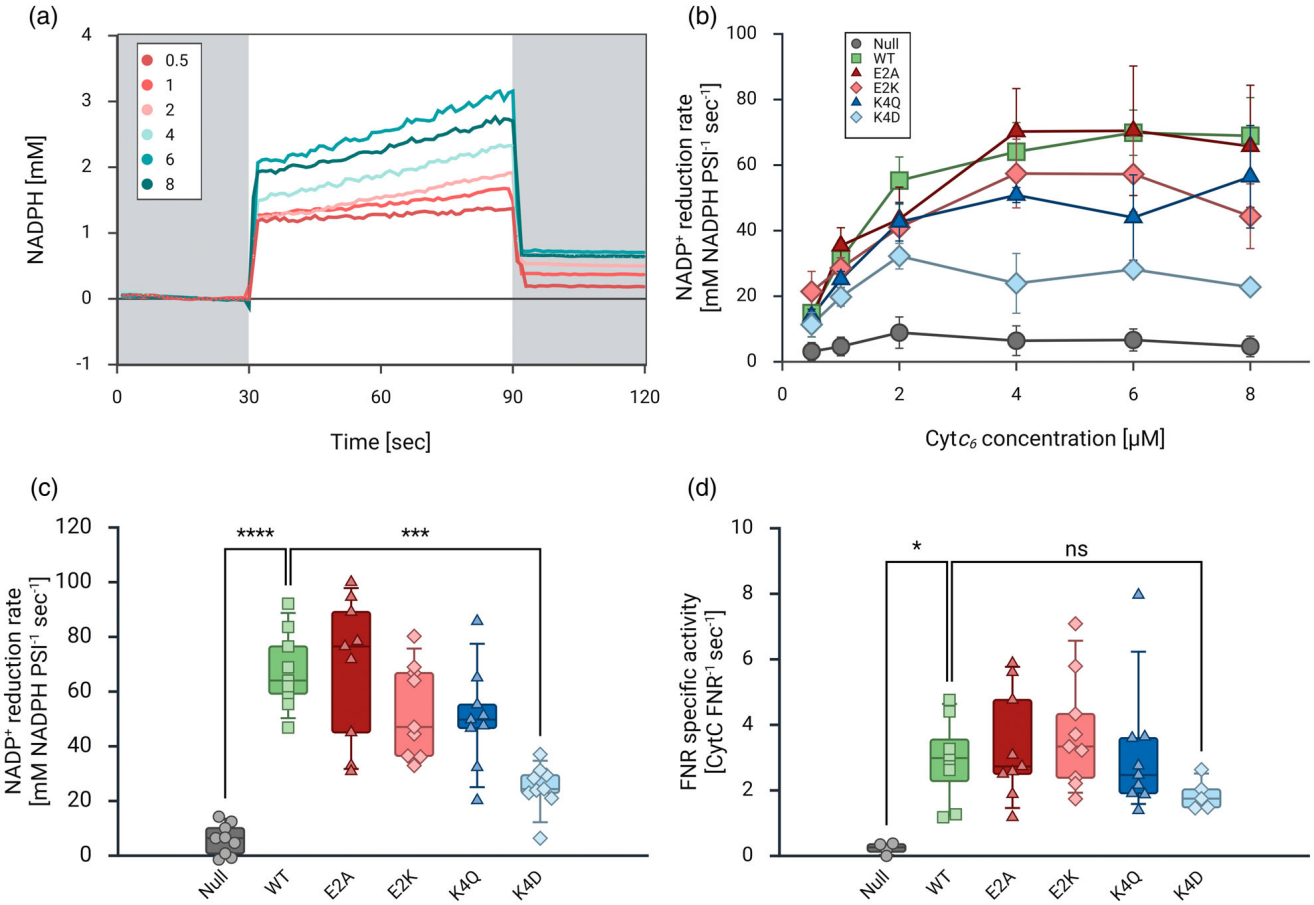
Kinetic analysis of FNR activity and functional validation of the PSI–FNR interface. (a) Light‐induced NADPH production monitored over 60 s (A_340_) in a reconstituted system containing isolated PSI, ferredoxin (FDX), and NADP^+^, with varying concentrations of the electron donor Cyt*c*_6_ (0.5–8 μM). (b) NADPH production rates as a function of Cyt*c*_6_ concentration in the absence (null, gray circles) or the presence of wild‐type FNR (WT, green squares); interface mutants were also tested: E2A (red triangles), E2K (red diamonds), K4Q (blue triangles), and K4D (blue diamonds). Data points represent average of three biological replicates; error bars represent standard error. (c) Steady‐state NADPH reduction rates at saturating Cyt*c*_6_ concentrations (4–8 μM). Box plots represent the median, interquartile range, and whiskers extending to the minimum/maximum values. (d) FDX‐dependent cytochrome *c* reduction (A_550_) as a control for intrinsic FNR catalytic activity. The lack of significant difference between WT and interface mutants confirms that observed kinetic changes in (c) are due to altered PSI binding rather than impaired enzymatic turnover. Statistical significance was determined by Welch's one‐way ANOVA followed by Games–Howell multiple comparisons test; *P < 0.05, ***P < 0.001, ****P < 0.0001; ns, not significant. Figure and statistical analysis were generated using BioRender.com.

**Figure 4:**
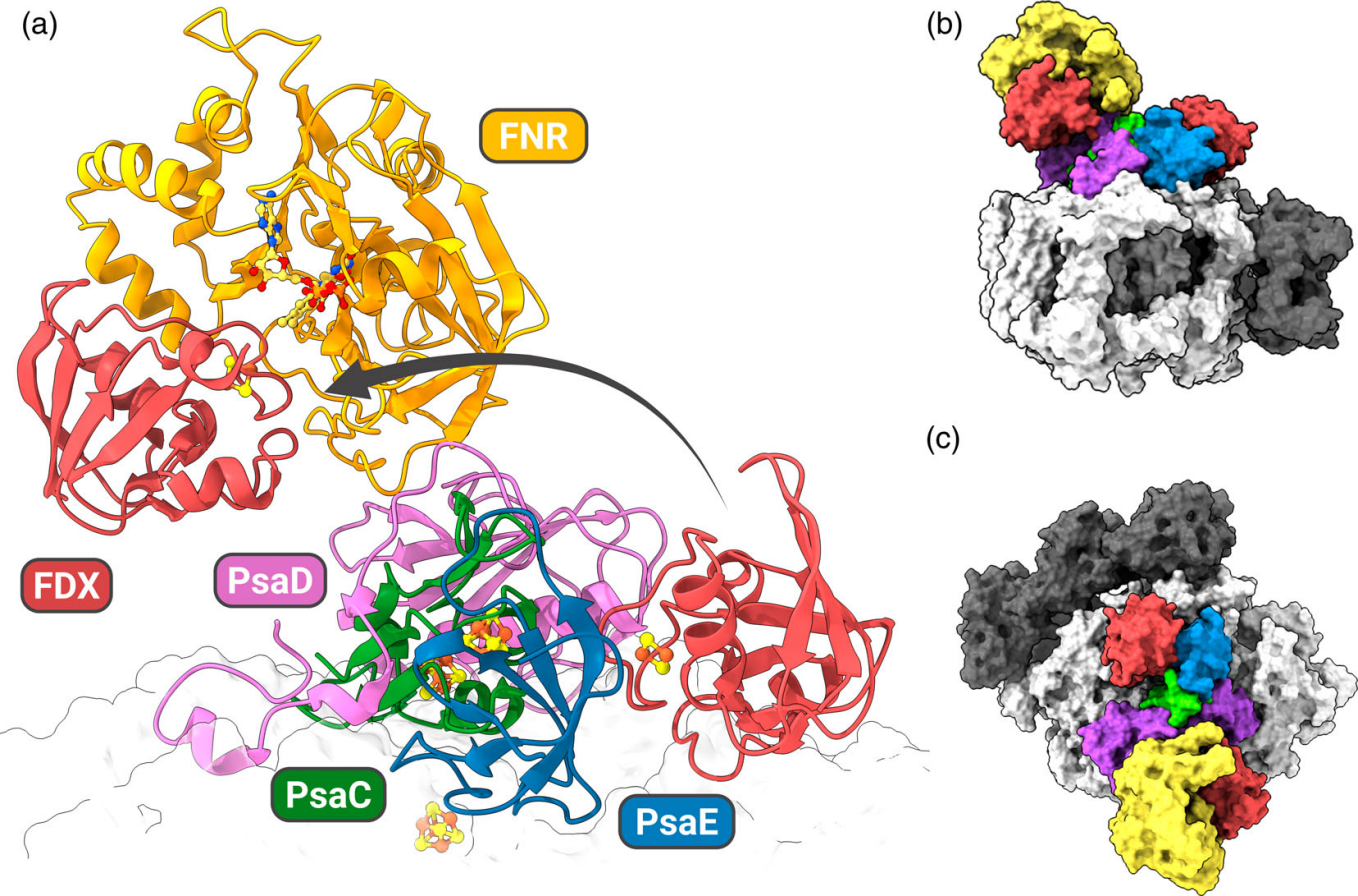
Steric compatibility of simultaneous FDX and FNR binding to the PSI stromal ridge. (a) Structural model of the FNR–FDX–PSI ternary assembly. Ribbon representation shows FNR (yellow) and FDX (red) docked onto the stromal subunits PsaC (green), PsaD (pink), and PsaE (blue). The gray arrow indicates the putative [4Fe‐4S] cluster‐mediated electron transfer pathway from PSI to FNR via FDX. (b, c) Molecular surface representations of the assembly in side view (b) and top‐down view (c). The distinct binding footprints of FNR and FDX demonstrate a lack of steric hindrance, suggesting that FNR recruitment to the PSI stromal ridge does not preclude FDX binding or turnover. The figure was generated using ChimeraX and BioRender.com.

To assess the functional relevance of the PSI–FNR complex interface as predicted by the docking model, site‐directed mutagenesis was employed on selected FNR residues. The K4 residue of FNR, which is potentially forming a salt bridge with PsaD:E91 and a conventional hydrogen bond with G90, was substituted by either a neutral (glutamine ‐ Q) or negatively charged (aspartate ‐ D) amino acids. Additionally, a second residue (E2), which is located near the salt bridge forming residue (K4) and which does not make any direct interaction with any residue of PSI in the predicted model (Figure 2), was also substituted with either K or A (alanine, neutral). Kinetic assay measuring the reduction of NADP^+^ to NADPH (Figure 3a) was conducted using isolated PSI and each of the described FNR mutants, which were purified using a recombinant expression system (Marco et al., 2019), namely, WT, E2A, E2K, K4Q, and K4D. To verify that the kinetics reflect the rate of FNR reduction, we designed the experiment to contain minimal ferredoxin (FDX) amounts (0.1 μM) and added increasing concentrations of Cyt*c*_*6*_, which was also recombinantly expressed using the protein sequence of *C. merolae* (UniProt entry nr: Q85FS2). The rates of NADPH formation were plotted using a linear fit (Figure 3b). The traces which showed no further increase in rate as a function of Cyt*c*_*6*_ concentration (meaning 4–8 μM) were averaged and compared using Welch's one‐way ANOVA with Games–Howell multiple comparisons test (Biorender.com). These results revealed that the charge switch on the salt bridge forming K4 residue resulted in a strong reduction of FNR activity, leading to a significantly decreased rates of NADP^+^ reduction as compared to the wild‐type (WT) FNR (Figure 3b,c). The other residue (E2) which does not seem to make any direct interaction in the predicted model, had no significant effect on NADP^+^ reduction kinetics (Figure 3c). The differential effects of K4D and K4Q mutations indicate that the electrostatic nature of K4 contributes to the productive PSI–FNR interaction. Conversely, the absence of significant effects in the E2 mutants suggest that the functional impact of charge alteration is position‐dependent within the PSI–FNR docking interface. Moreover, to see whether the mutations could interfere with the binding of FDX to FNR, the cytochrome c reduction assay (FNR specific activity) was also performed (Figure 3d). This assay did not involve the PSI in the reaction mixture as compared with the above NADP^+^ reduction assay and allows us to see the interaction of FDX with FNR. No significant difference was observed between the WT and all the other mutants, suggesting that FDX can bind the FNR for electron delivery without any significant reduction in rate (Figure 3d), which further strengthens our predicted FNR–PSI model. Figure 4 reveals that the binding of FNR to PSI is in such a way that it does not clash with the binding of FDX to PSI. Moreover, the FDX binding pocket on FNR is also freely accessible to FDX.

## DISCUSSION

Here, we generated an interactome map for PSI complex of *C. merolae*, a member of the red lineage. Most importantly, our XL‐MS data indicated a direct binding of FNR to PSI via electrostatic interactions between FNR and PsaD. The HADDOCK2.4 mediated modeling revealed a PSI–FNR complex, in which the K4 residue of FNR forms a salt bridge with PsaD:E91 and a conventional hydrogen bond with PsaD:G90. Substitution of FNR residue K4 by Q or D markedly impaired NADP^+^ reduction compared with the WT FNR (Figure 3b,c), supporting that FNR binding to PSI is required for efficient FNR and FDX‐dependent NADP^+^ photoreduction. In contrast, NADPH‐driven reduction of horse heart cyt*c* was unaffected, implying that binding and electron transfer between FNR K4Q/K4D and FDX are not compromised. These results strongly suggest that FNR binds to the stromal side of PSI in an orientation that facilitates efficient electron transfer from FDX to form NADPH.

Such a stable, functional PSI–FNR binding in a protein complex has been demonstrated in *C. reinhardtii*, where an isolated PSI–FNR complex supported competent NADP^+^ photoreduction in the presence of FDX without soluble FNR (Mosebach et al., 2017; Takahashi et al., 2014), and loss of the CEF factors PGRL1 and PGR5 diminished both thylakoid FNR association and NADP^+^ photoreduction, indicating a link between FNR membrane binding and CEF. In vascular plants, FNR tethering to TROL and Tic62 is likewise required for efficient P700 oxidation during dark‐to‐light transitions, and its disruption accelerates PSI photoinactivation and impairs CEF, underscoring the importance of FNR localization for regulating photosynthetic electron flow (Kramer et al., 2021; Rodriguez‐Heredia et al., 2022). Sustained P700 oxidation is required to prevent PSI photoinhibition (Shimakawa et al., 2016), and FNR recruitment to PSI may enhance this process while promoting efficient two‐electron reduction of NADP^+^, thereby shortening semiquinone lifetime and limiting O_2_‐dependent side reactions during LEF (Bergner et al., 2015; Takahashi et al., 2014). Moreover, FNR abundance and localization influence ROS production (Kozuleva et al., 2016), and its association with Cyt*b*_*6*_ *f* has been proposed to stimulate CEF, positioning FNR relocation as a key regulator of photosynthetic electron partitioning (Goss & Hanke, 2014; Joliot & Johnson, 2011). Notably, tethering FNR to PSI via PSAF in *C. reinhardtii* (Emrich‐Mills et al., 2025), generated chimeric mutants with reduced photosynthetic growth and LEF due to slower NADPH reduction and increased PSI acceptor‐side limitation, while enhanced ΔpH, NPQ, and CEF indicated a shift from LEF and CO_2_ fixation toward CEF. Structural superposition (Figure 4) shows that FNR docks on the stromal side of PSI without obstructing FDX binding, supporting efficient electron transfer to PSI‐bound FNR and potentially limiting semiquinone lifetime, O_2_ side reactions, and ROS formation. In contrast, tethering FNR to PSAF is likely to constrain its positioning and reduce the efficiency of FDX–FNR interaction and electron transfer to form NADPH. Thus, underpinning the importance of correct FNR binding to PSI for efficient formation of NADPH.

While the FNR–PSI interaction represents the primary focus of this study, our cross‐linking dataset also revealed additional interactions involving PSI subunits. Among these, we identified a cross‐link between apcA and PsaD, suggesting a potential physical association between PBS and PSI. Several studies have reported that a part of the energy that PBS absorbs can be delivered to PSI (Dong et al., 2009; Glazer et al., 1994; Mullineaux, 1992; Rakhimberdieva et al., 2001). In *C. merolae*, the energy transfer from PBS to PSI has been observed by cultivating cells under different monochromatic lights (Ueno et al., 2015). Moreover, previous biochemical, spectroscopic, and mass spectrometric measurements have indicated that PBS is found attached to the isolated PSI particles in *C. merolae* and it was revealed that this PSI‐specific PBS subcomplex can transfer excitation energy to PSI (Busch et al., 2010). Interestingly, this complex was proposed to contain phycocyanin and the linker cpcG while lacking allophycocyanin (Busch et al., 2010). Similarly, in *Anabaena* sp. PCC 7120, a PSI‐specific rod‐shaped PBS composed of phycocyanin and the linker CpcL, but devoid of allophycocyanin and CpcGs, was also observed by biochemical, spectroscopic, and low‐resolution EM approaches (Watanabe et al., 2014). Contrastingly, our data reveal a direct cross‐link between the allophycocyanin core subunit apcA and PSI, suggesting that the PBS core itself may participate in interacting with PSI.

In the red algae as well as cyanobacteria, the PBS cores are typically viewed as antennae that are mainly associated with PSII, with energy transfer to PSI occurring via a mechanism known as “spillover,” that is, redistribution of excitation from an overexcited PSII/PBS domain to a nearby PSI. Recent cryo‐EM structure of PBS–PSII–PSI–LHC megacomplex in the red alga *Porphyridium purpureum* reveals direct structural evidence that supports such spillover‐type energy redistribution (You et al., 2023). Consistently, XL‐MS has already been employed before to identify potential interaction sites in cyanobacterial PBS–PSII–PSI complex (Liu et al., 2013). Nevertheless, spillover may not represent the only mechanism for PBS‐mediated excitation transfer to PSI. In most cyanobacterial species, the core component apcD has been revealed to be necessary for state transitions (Calzadilla et al., 2019; Dong et al., 2009; Dong & Zhao, 2008). Without apcD, the PBS‐derived light‐harvesting cross‐section for PSI was markedly reduced (Calzadilla et al., 2019; Dong et al., 2009). Recently, it was also observed that, in *Synechocystis*, the deletion of apcG resulted in reduced fluorescence of PSI and further suggested that apcG phosphorylation play a role in energy transfer regulation from PSII to PSI (Espinoza‐Corral et al., 2024). Additionally, in the mesophilic red algae *Rhodella violacea* and *Porphyridium cruentum*, the mobility of PBS was also observed (Kaňa et al., 2014), further supporting the dynamic nature of interaction between PBS and photosystem. Despite these advances, the molecular details of energy transfer from PBS to PSI remain incompletely understood. It is usually difficult to obtain the intact assemblies using single particle analysis due to dissociation of some complexes that are loosely associated. Taken together, previous spectroscopic and biochemical studies, together with our cross‐linking data, support the idea that PBS can dynamically interact with PSI in *C. merolae* to redistribute excitation energy in response to changing environmental conditions.

Clearly, the FNR:K94–PsaD:K108 and apcA:K27–PsaD:K108 cross‐links overlap, indicating that PsaD is involved in binding of FNR and PBS. Interestingly, FNR has been found associated with PBSs in cyanobacteria such as *Synechocystis* and *Synechococcus* (Gómez‐Lojero et al., 2003; Van Thor, 1999). In *Synechocystis* sp. PCC 6803, two distinct FNR isoforms are produced from a single *petH* gene via an internal ribosome entry site: the larger FNR(L) associates with PBSs and functions as an NADP^+^ reductase, whereas the smaller FNR(S), which accumulates under heterotrophic conditions, likely acts as an NADPH oxidase (Thomas et al., 2006). Yet, during linear electron transport, a PsaE‐dependent transient PSI–ferredoxin–FNR ternary complex is suggested to form in cyanobacteria (Van Thor et al., 1999). Thus, direct binding of FNR to PSI is also suggested for cyanobacteria, indicating that PBS and FNR binding to PSI might not be mutually exclusive. On the other hand, if PBS binding to PSI inhibits FNR binding, FDX can still be reduced and released to transfer electrons to FNR. Thus, PBS and FNR binding to PSI may either occur simultaneously or compete for overlapping sites, although electron transfer could still proceed via soluble FDX if PBS association transiently restricts direct FNR docking.

## MATERIAL AND METHODS

### Culture cultivation

*C. merolae* 10D was kindly provided by Professor Tsuneyoshi Kuroiwa (Department of Life Science, Rikkyo University, Japan) and cultivated in Allen medium (2×) as previously described (Minoda et al., 2004) at either 25 or 42°C with constant shaking (100 rpm) and continuous light (approximately 60 μmol photons m^−2^ sec^−1^).

### Thylakoid membranes isolation and cross‐linking

Thylakoid membranes were isolated from the *C. merolae* cultures grown at either 25 or 42°C using a modified protocol as described before (Chua et al., 1975), by first breaking the cells with the help of a self‐made bio‐nebulizer (20 psi pressure). The cells were passed through the nebulizer two times for breaking. Broken cells were then subjected to centrifugation (20 000 rpm, 10 min, 4°C; JA 25.50 rotor, Beckman Coulter), and the pellet, with the help of a homogenizer/potter, was resuspended carefully in a buffer containing 5 mM HEPES‐KOH (pH 7.5), 10 mM EDTA, 1.8 M sucrose, 5 mM ACA and 1 mM BA. The resuspended material (12 ml) was poured into SW 32 tubes and subsequently other buffers (equal amounts, i.e., 12 ml) with varying sucrose concentration (1.3 M sucrose and 0.5 M sucrose plus 5 mM HEPES‐KOH pH 7.5, 10 mM EDTA, 5 mM ACA, 1 mM BA) were carefully added on top to make a sucrose density step gradients, followed by ultracentrifugation (24 000 rpm, 1 h 20 min, 4°C; SW 32 Ti rotor, Beckman Coulter) to extract the thylakoid membranes, which were later on harvested from the gradient interphases with the help of a Pasteur pipet and diluted three to four times with a buffer containing 5 mM HEPES (pH 7.5). The resulting material was then centrifuged again (21 500 rpm, 20 min, 4°C; JA 25.50 rotor, Beckman Coulter). The thylakoids were either used directly for cross‐linking and solubilization purposes or treated with NaBr, as described previously (Busch et al., 2010; Nikolova et al., 2017), prior to cross‐linking and solubilization. For cross‐linking reaction, 0.5 mm DSS (Creative Molecules), which was first dissolved in DMSO, was added to isolated thylakoid membranes (0.2 mg Chl ml^−1^) and the reaction was allowed to proceed for 30 min in the dark at room temperature. For the quenching reaction, Tris–HCL (pH 7.5) was added to a final concentration of 50 mM.

### Thylakoid solubilization and SDG ultracentrifugation

For solubilization of thylakoids, three different detergents, namely, n‐Dodecyl α‐D‐maltoside (α‐DM), n‐dodecyl β‐D maltoside (β‐DM), and octyl glucoside (OG) were employed. For solubilization using α‐DM, 50 μl of 10% α‐DM was added to the isolated thylakoids (200 μg ml^−1^), followed by incubation in the dark for 5 min at 4°C. For OG, 96 μl of 10% OG was added to thylakoids (200 μg ml^−1^), followed by incubation for 30 min in the dark at 4°C. After solubilization, the insolubilized materials were removed using centrifugation (full speed, 5 min, 4°C; Eppendorf centrifuge) and the solubilized thylakoids were loaded on top of SDGs (0.5–1.3 M sucrose from top to bottom), followed by ultracentrifugation (33 000 rpm, 16 h, 4°C; SW 41 Ti rotor, Beckman Coulter) to isolate the different complexes. SDGs were then fractionated by puncturing the gradient at the bottom.

### Photosystem I purification

The photosystem I (PSI) containing fractions were collected from α‐DM treated samples and loaded onto a column (Toyopearl) equilibrated with 10 column volume (CV) of a buffer encompassing 0.05% α‐DM and 20 mM Tricine pH 8.0. Washing was done in two steps with increasing conc. of KCl, that is, wash I (5 mM KCl; 10 CV) and wash II (10 mM KCl; 10 CV), followed by elution at 25 mM KCl, 50 mM KCl, and 100 mM KCl with 10–15 ml each. Subsequently, 77 K spectroscopy was performed to collect the PSI containing fractions which were then enriched using a 100 kDa centricon and washed several times with the equilibration buffer to ensure purity of the PSI samples. The PSI samples were then measured and aliquots containing 28 μg Chl in 40 μl were prepared and stored at −80°C after flash freezing in liquid nitrogen for further analysis.

### FDX purification

FDX (plasmid: Pet21b:FDX:TEVc:MBP:His6) and Tobacco Etch Virus protease (plasmid: pMHTDelta238) heterologously expressed in *Escherichia coli* were a kind gift from Prof. Dr. Iftach Yacoby from Tel‐Aviv University. Expression and purification were conducted as described in Marco et al. (2018).

### Recombinant Cyt*c*_6_ purification

Recombinant Cyt*c*_6_ plasmids were synthesized and ligated as described in Ogawa et al. (2026). The pre‐cultures of *E. coli* BL21 Star::pEC86::pET22‐b were inoculated using LB media containing ampicillin and chloramphenicol and incubated for 12 h at 34°C and 100 rpm. The main culture was then inoculated using 1 L of LB media containing ampicillin and chloramphenicol plus the above preculture and grown at 37°C at 100 rpm until the OD_600_ reached 0.5–0.7, which was subsequently induced by adding IPTG (1 mM) and incubated again at 30°C and 100 rpm overnight. The main culture was then harvested by centrifugation (5000 rpm, 5 min, 4°C; JLA 16.250 rotor, Beckman Coulter). The resulting pellet was then resuspended in Cyt*c*_6_ lysis buffer (20 ml) containing 1 mM PMSF and sonicated twice (nine impulses with a 10 sec duration each) and centrifuged (10 000× ***g***, 30 min, 4°C). The resulting supernatant was loaded onto a pre‐equilibrated DEAE column. During loading, 1 mM ascorbate was also added. Subsequently, the column was washed with two to four CV of wash buffer and the Cyt*c*_6_ was then eluted using 15 ml of Cyt*c*_6_ elution buffer, followed by concentration of the eluate in a 3 kDa centricon to a final volume of around 1 ml and then washed with 15–20 ml of a buffer containing 10 mM KCl and 20 mM Tricine (pH 7.8). The concentrated eluate containing Cyt*c*_6_ was then subjected to SEC two times using Superdex 65 10/300 GL column coupled to ÄKTA pure FPLC system. Fractions of interest were collected, and the spectra measurements were recorded afterwards. Purity of the samples was assessed by evaluating A_420_/_A280_ and A_552_/A_280_ ratios. Subsequently, the samples were concentrated again using 3 kDa centricon to a volume of 150–200 μl and another spectra measurement was recorded to find the concentration using c = A_552_ ε^−1^d^−1^ based on Beer–Lambert Law with the Cyt*c*_6_‐specific extinction coefficient ε_552_ = 24.8 mM^−1^ cm^−1^ and d = 1 cm. Aliquots containing 50 μM Cyt*c*_6_ were prepared, frozen in liquid nitrogen and stored at −80°C for further analysis.

### Recombinant *C. merolae* FNR assembly and purification

The protocol for assembly of recombinant proteins was based on Kuhlgert et al. (2012). The synthetic DNA sequences of *C. merolae* FNR gene, flanked by complementary BsaI restriction sites sequences, were ordered from Eurofins Genomics and sub‐cloned into the plasmid Pet21b:FDX:TEVc:MBP:His6 using the GoldenGate cloning technique; the target plasmid was amplified with matching BsaI sites and without the native FDX sequence, which was replaced by our synthetic gene sequence. 20 fmol of vector was used with 20 fmol synthetic DNA for three successive rounds of digestion (BsaI, 37°C, 10 min) and ligation (T4 DNA ligase, 16°C, 10 min) in the same mix. TOP10 *E. coli* cells (NEB) were then transformed with the ligation products for vector amplification, screening, and sequencing. About 50 ng of vector were then used to transform chemically competent *E. coli* BL21(DE3) cells (NEB) for protein expression. Expression and purification were conducted as described in Marco et al. (2019).

Site‐directed mutagenesis for the different *C. merolae* FNR variants was based on Zheng et al. (2004). Briefly, the WT plasmid was amplified via PCR using NEB Q5 High‐Fidelity 2× Master Mix in 25 μl reaction volumes, containing 10 ng DNA template and 0.5 μm of specific primer pairs carrying the new codon for each PC variant (D169N mutation: AAC; D169K mutation: AAA; K4D: GAT; K4Q: CAG; E2A: GCG; E2K: AAA); the PCR protocol was set according to the manufacturer's instructions (amplification was done for 25 cycles with 100 s elongation time each), and annealing temperatures were calculated according to the online NEB Tm calculator. After amplification, 1 μl of PCR product was incubated at RT for 10 min with NEB 10× KLD Enzyme Mix in a 10 μl reaction volume for removal of the original WT DNA template and for ligation of the PCR products, before being used to transform TOP10 *E. coli* cells for plasmid isolation and Sanger sequencing (Eurofins Genomics). Plasmids with the correct sequence were used to transform *E. coli* BL21(DE3) cells (NEB) for recombinant protein production as described above.

### Light‐dependent NADPH production assay

Light‐dependent NADPH production mediated by PSI and FNR was based on Mosebach et al. (2017), using samples containing isolated PSI particles (17.5 μg Chl ml^−1^), 30 mM MOPS (pH 7.0), 0.01% tPCCαM, 2.5 mM MgCl_2_, 10 mM Na‐Ascorbate, 0.1 μM FDX, 2.5 mM NADP+, 0.5 μM Cyt*c*_6_, and 0.1 μM FNR. NADP+ photoreduction was measured via following absorption changes at 340 nm during 60 sec of saturating illumination with a white halogen lamp. Cyt*c*_6_ concentration was increased in between each measurement as described in Figure 3(b).

### FNR‐cytochrome c reductase activity assay

Activity assays were based on Fisher et al. (2019), by following absorption changes at 550 nm (and at 542 nm for correction) during 180 sec. The reaction medium consisted of 10 mM HEPES (pH 7.6), 5 mM MgCl_2_, 1 μM FDX, 20 μM equine cytochrome c, and 1 μM FNR; the reaction was initiated by the addition of 100 μM NADPH.

### LC–MS/MS analysis

LC–MS/MS analysis of the cross‐linked peptide samples was performed as previously described (Mosebach et al., 2024). The LC–MS/MS setup comprised of nano HPLC system (Ultimate 3000, Thermo Fisher Scientific) coupled to a Q Exactive Plus mass spectrometer (Thermo Fisher Scientific) via a nano‐electrospray ionization (ESI) source (Thermo Fisher Scientific) was employed for the analysis. Resuspension of samples was done in solvent A1 (containing trifluoroacetic acid at 0.05%, acetonitrile at 4%, as well as ultrapure water) and afterwards loaded on to a C18 trap column at a flow rate of 7.5 μl min^−1^ for 5 min. Subsequently, the peptides, in a backflush mode, were then eluted onto a separation C18 column and separated at a flow rate of 300 nL min^−1^. The details and dimensions of C18 columns are given in Mosebach et al. (2024). Peptide separation was performed using the following eluents: solvent A comprising of 0.1% formic acid in ultrapure water and, solvent B consisting of 80% acetonitrile and 0.1% formic acid in ultrapure water. Peptides were eluted using a stepwise gradient starting from 2.5% of solvent B, increasing to 7.5% over 4 min, followed by a linear increase to 40% over 24 min of solvent B. After a hold at 40% for 3 min, the solvent B was increased to 99% for 3 min and then maintained at 99% for another 10 min.

Mass spectra were recorded in the m/z range of 400–2000 at 70 000 resolution (FWHM at 200 m/z) with internal lock mass calibration. Fragmentation spectra (MS2) were collected at 35 000 resolution (FWHM at 200 m/z) in a data‐dependent manner, in which the 12 most intense ions were selected for fragmentation from the full scan. Fragmentation of ions was performed by HCD (higher‐energy c‐trap dissociation) using a normalized collision energy of 27 and an isolation window of two m/z. The automatic gain control targets were set to 1e6 and 1e5 for MS full scans (MS1) and MS2, respectively, with a maximum injection time of 50 and 100 ms for MS1 and MS2, respectively. For MS2, the intensity threshold was 1e4. The following ions were excluded from fragmentation: the ions with unassigned charge states or charge states 1 or 2, or charge states 8 or higher.

### MS data analysis

MS raw files were searched against a curated database of chloroplast proteins encompassing predominantly PSI, PSII, Cyt*b*_6_ *f*, ATP synthase, LHC and associated proteins (Supplementary File 1), retrieved from UniProt. DSS‐cross‐linked peptides were identified using five complementary search engines: XiSearch 1.7.6.7 (Matzinger et al., 2022), MaxQuant 2.0.3.0 (Tyanova et al., 2016) and pLink 2 (Chen et al., 2019). For XiSearch analyses, raw spectral data were converted to mgf format using MSConvert 3.0.23326 (Mosebach et al., 2024). Searches were performed using each algorithm's default parameters with the following unified settings: precursor and fragment ion mass tolerances of 10 and 15 ppm, respectively; minimum peptide length of 5 amino acids; N‐terminal acetylation and methionine oxidation as variable modifications. Cross‐linked peptide identifications were filtered at a false discovery rate of 5%. The mass spectrometry proteomics data have been deposited to the ProteomeXchange Consortium via the PRIDE (Perez‐Riverol et al., 2025) partner repository with the dataset identifier PXD079525.

### Structural modeling

For the modeling of FNR, the transit peptide was first predicted with TargetP 2.0 (Almagro Armenteros et al., 2019) and then removed from the final sequence of FNR before structural prediction with AlphaFold 3 (Abramson et al., 2024). The sequence for FNR was retrieved from UniProt (entry nr: M1V6N7). The predicted model was then docked to the available PSI structure of *C. merolae* using the HADDOCK2.4 server (Honorato et al., 2024), using cross‐linking data as distance restraints. The final figures regarding protein structures were then generated using ChimeraX, and the interactions were further observed using the BIOVIA Discovery Studio Visualizer (BIOVIA, Dassault Systèmes, Discovery Studio Visualizer, v21.1.0.20298, San Diego: Dassault Systèmes, 2021). Similarly, for the interactome modeling, Cytoscape was employed (Shannon et al., 2003).

### PsaE sequence comparison

Sequence alignment of PsaE was conducted as described in Milrad et al., (2025). All available sequences were retrieved from UniProt using search‐word “psaE.” 29 146 results were retrieved and filtered to contain either the gene or protein name: “psaE.” Out of the entire collection, 1310 sequences were adequate and were organized based on taxonomic lineage. Residue alignment was based on conserved regions and structural functionality. Finally, sequences that did not meet any resemblance or were attributed to a non‐photosynthetic origin were filtered out, resulting in 1236 sequences from most known photosynthetic lineages (Supplementary File 2).

## AUTHOR CONTRIBUTIONS

Research design was conducted by M.Y., Y.M., A.V‐M., and M.H. *in vivo* cross‐linking experiments and sample preparation were conducted by M.Y. and K.Z. Mass‐spectromerty studies and analysis were conducted by M.S. Protein construction, purifications, and kinetic experimentation were conducted by M.Y., Y.M., S.W., and A.V‐M. Structural modeling was conducted by M.Y., and Y.M. The manuscript was written by M.Y., Y.M., and M.H.

## CONFLICT OF INTEREST

None declared.

## Supporting information


**Figure S1.** Expanded XL‐MS interactome of the photosynthetic machinery. Global protein–protein interaction network showing detected cross‐links within and between major thylakoid membrane protein complexes. Subunit clusters are color‐coded by functional assembly: phycobilisome (blue), Photosystem I (green), Photosystem II (yellow), ATP synthase (purple), and the Cytochrome *b*_*6*_ *f* complex (wheat). The FNR subunit (orange) is shown interacting specifically with the PsaD subunit of PSI. Solid lines indicate intracomplex cross‐links, while dashed gray lines highlight intercomplex interactions, including the connectivity between the phycobilisome (ApcA) and both PSI (PsaD) and PSII (PsbC). Nodes represent individual protein subunits identified by mass spectrometry.

**Figure S2.** Two‐dimensional representation of the FNR–PsaD docking interface. Interactions between FNR (Chain B) and PsaD (Chain A) were analyzed using LigPlot+. Hydrogen bonds are indicated by dashed green lines, with donor–acceptor distances labeled in Ȧ. Hydrophobic contacts are represented by red and pink arcs with spokes radiating toward the atoms involved in the interaction. This schematic highlights the stabilization of the complex through key residues, including FNR‐K4 and PsaD‐E91, which were targeted for functional validation in Figure 3.

**Figure S3.** Multiple sequence alignment of the PsaE subunit across diverse photosynthetic lineages. Comprehensive alignment of PsaE amino acid sequences retrieved from the UniProt database, categorized by taxonomic group (indicated by colored bars on the left). The alignment highlights the presence of a C‐terminal extension (the “loop” characterized in Figure 1) that is specifically enriched in red algae (Rhodophyta). Amino acids are color‐coded according to their physicochemical properties. Note the high degree of conservation in the core domain contrasted with the lineage‐specific sequence variation in the C‐terminal loop region, which facilitates specific interactions with peripheral complexes like the phycobilisome.

**Supplementary File 1.** MS results cross‐links.

**Supplementary File 2.** Sequence align‐PsaE.

**Supplementary File 3.** PSI–FNR_RCm‐Model.

## Data Availability

All data will be available online once accepted. Upon submission, the authors attached additional files that include all raw data and analysis. For any additional request, please contact the corresponding author
