# Supplementary figures and images for: Structural proteomics reveals the functional docking interface of ferredoxin‐NADP + reductase on photosystem I in the red alga Cyanidioschyzon merolae
Source: Plant J. 2026 Jul 1;127(1):e71017. doi: 10.1111/tpj.71017 (PMC13322767; doi:10.1111/tpj.71017)

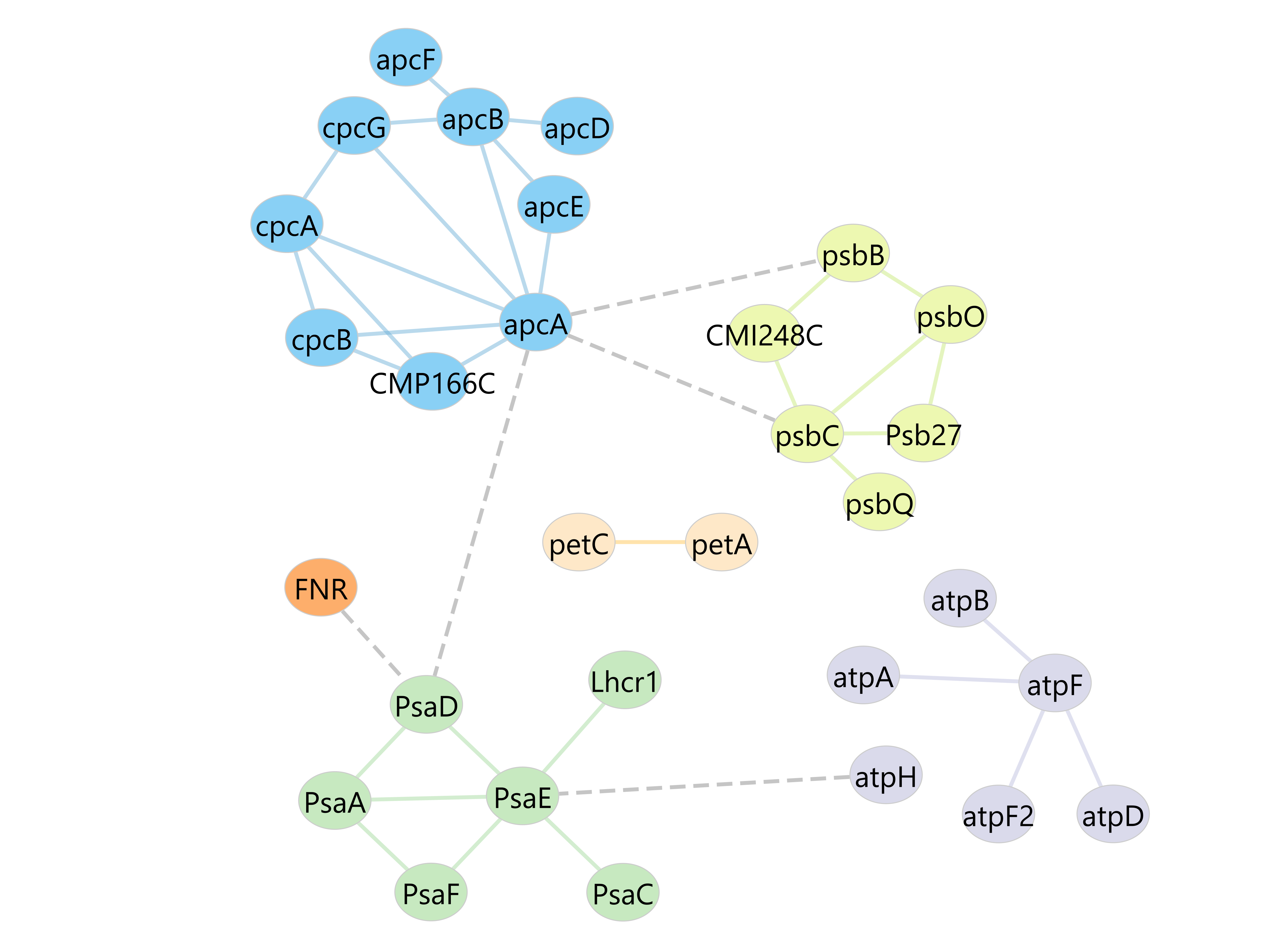

Supplement: Supplementary file 1 — Figure S1. Expanded XL‐MS interactome of the photosynthetic machinery. Global protein–protein interaction network showing detected cross‐links within and between major thylakoid membrane protein complexes. Subunit clusters are color‐coded by functional assembly: phycobilisome (blue), Photosystem I (green), Photosystem II (yellow), ATP synthase (purple), and the Cytochrome b 6 f complex (wheat). The FNR subunit (orange) is shown interacting specifically with the PsaD subunit of PSI. Solid lines indicate intracomplex cross‐links, while dashed gray lines highlight intercomplex interactions, including the connectivity between the phycobilisome (ApcA) and both PSI (PsaD) and PSII (PsbC). Nodes represent individual protein subunits identified by mass spectrometry. [file TPJ-127-0-s005.tif]

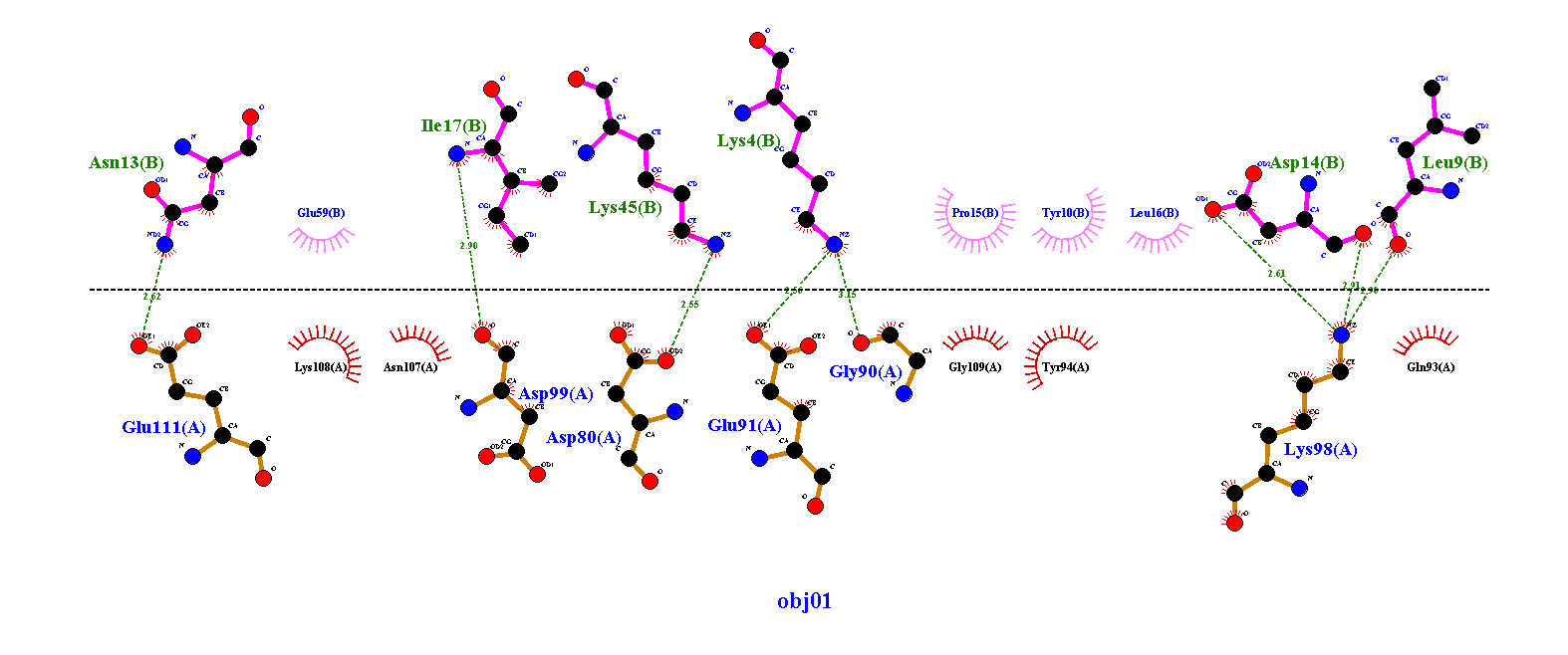

Supplement: Supplementary file 2 — Figure S2. Two‐dimensional representation of the FNR–PsaD docking interface. Interactions between FNR (Chain B) and PsaD (Chain A) were analyzed using LigPlot+. Hydrogen bonds are indicated by dashed green lines, with donor–acceptor distances labeled in Ȧ. Hydrophobic contacts are represented by red and pink arcs with spokes radiating toward the atoms involved in the interaction. This schematic highlights the stabilization of the complex through key residues, including FNR‐K4 and PsaD‐E91, which were targeted for functional validation in Figure 3. [file TPJ-127-0-s003.tif]
